# Charting a course for global progress in PIDs by 2030 — proceedings from the IPOPI global multi-stakeholders’ summit (September 2023)

**DOI:** 10.3389/fimmu.2024.1430678

**Published:** 2024-06-27

**Authors:** Samya Van Coillie, Johan Prévot, Silvia Sánchez-Ramón, David M. Lowe, Michael Borg, Brigitte Autran, Gesmar Segundo, Antonio Pecoraro, Nicolas Garcelon, Cornelis Boersma, Susana L. Silva, Jose Drabwell, Isabella Quinti, Isabelle Meyts, Adli Ali, Siobhan O. Burns, Martin van Hagen, Martine Pergent, Nizar Mahlaoui

**Affiliations:** ^1^ International Patient Organisation for Primary Immunodeficiencies (IPOPI), Brussels, Belgium; ^2^ Department of Clinical Immunology, Health Research Institute of the Hospital Clínico San Carlos/Fundación para la Investigación Biomédica del Hospital Clínico San Carlos (IML and IdISSC), Health Research Institute of the Hospital Clínico San Carlos (IdISSC), Madrid, Spain; ^3^ Department of Immunology, Royal Free London National Heath System (NHS) Foundation Trust, London, United Kingdom; ^4^ Institute of Immunity and Transplantation, University College London, London, United Kingdom; ^5^ Department of Infection Control & Sterile Services, Mater Dei Hospital, Msida, Malta; ^6^ Sorbonne-Université, Cimi-Paris, Institut national de la santé et de la recherche médicale (INSERM) U1135, centre national de la recherche scientifique (CNRS) ERL8255, Université Pierre et Marie Curie Centre de Recherche n°7 (UPMC CR7), Paris, France; ^7^ Departamento de Pediatra, Universidade Federal de Uberlândia, Uberlandia, MG, Brazil; ^8^ Transfusion Medicine Unit, Azienda Sanitaria Territoriale, Ascoli Piceno, Italy; ^9^ Université de Paris, Imagine Institute, Data Science Platform, Institut national de la santé et de la recherche médicale Unité Mixte de Recherche (INSERM UMR) 1163, Paris, France; ^10^ Health-Ecore B.V., Zeist, Netherlands; ^11^ Unit of Global Health, Department of Health Sciences, University Medical Center Groningen (UMCG), University of Groningen, Groningen, Netherlands; ^12^ Department of Management Sciences, Open University, Heerlen, Netherlands; ^13^ Serviço de Imunoalergologia, Unidade Local de Saúde de Santa Maria, Lisbon, Portugal; ^14^ Instituto de Medicina Molecular João Lobo Antunes, Faculdade de Medicina, Universidade de Lisboa, Lisbon, Portugal; ^15^ Department of Molecular Medicine, Sapienza University of Rome, Rome, Italy; ^16^ Department of Pediatrics, University Hospitals Leuven, Department of Microbiology, Immunology and Transplantation, Katholieke Universiteit (KU) Leuven, Leuven, Belgium; ^17^ Department of Paediatrics, Faculty of Medicine, Universiti Kebangsaan Malaysia, Kuala Lumpur, Malaysia; ^18^ Hospital Tunku Ampuan Besar Tuanku Aishah Rohani, Universiti Kebangsaan Malaysia (UKM) Specialist Children’s Hospital, Universiti Kebangsaan Malaysia, Kuala Lumpur, Malaysia; ^19^ Department of Internal Medicine, Division of Allergy & Clinical Immunology, Erasmus University Medical Center Rotterdam, Rotterdam, Netherlands; ^20^ Department of Immunology, Erasmus University Medical Center Rotterdam, Rotterdam, Netherlands; ^21^ Pediatric Hematology-Immunology and Rheumatology Unit, Necker-Enfants malades University Hospital, Assistance Publique-Hôpitaux de Paris (AP-HP), Paris, France; ^22^ French National Reference Center for Primary Immune Deficiencies (CEREDIH), Necker-Enfants malades University Hospital, Assistance Publique-Hôpitaux de Paris (AP-HP), Paris, France

**Keywords:** primary immunodeficiencies (PID), rare diseases (RD), immunoglobulin replacement therapy (IGRT), targeted therapies, pandemic preparedness, antimicrobial resistance (AMR), patient-reported outcome measures (PROMs), artificial intelligence (AI)

## Abstract

The International Patient Organisation for Primary Immunodeficiencies (IPOPI) held its second Global Multi-Stakeholders’ Summit, an annual stimulating and forward-thinking meeting uniting experts to anticipate pivotal upcoming challenges and opportunities in the field of primary immunodeficiency (PID). The 2023 summit focused on three key identified discussion points: (i) How can immunoglobulin (Ig) therapy meet future personalized patient needs? (ii) Pandemic preparedness: what’s next for public health and potential challenges for the PID community? (iii) Diagnosing PIDs in 2030: what needs to happen to diagnose better and to diagnose more? Clinician-Scientists, patient representatives and other stakeholders explored avenues to improve Ig therapy through mechanistic insights and tailored Ig preparations/products according to patient-specific needs and local exposure to infectious agents, amongst others. Urgency for pandemic preparedness was discussed, as was the threat of shortage of antibiotics and increasing antimicrobial resistance, emphasizing the need for representation of PID patients and other vulnerable populations throughout crisis and care management. Discussion also covered the complexities of PID diagnosis, addressing issues such as global diagnostic disparities, the integration of patient-reported outcome measures, and the potential of artificial intelligence to increase PID diagnosis rates and to enhance diagnostic precision. These proceedings outline the outcomes and recommendations arising from the 2023 IPOPI Global Multi-Stakeholders’ Summit, offering valuable insights to inform future strategies in PID management and care. Integral to this initiative is its role in fostering collaborative efforts among stakeholders to prepare for the multiple challenges facing the global PID community.

## Introduction

The primary immunodeficiency (PID) field has evolved over the last decade owing to considerable advances in the identification of novel PID gene variants and their diagnosis, improved knowledge and understanding of the often-complex pathogenesis involved, and increased availability and optimization of treatment modalities. PIDs, also referred to as “Inborn Errors of Immunity” (IEI) are a heterogeneous group of disorders including infectious, autoimmune, autoinflammatory and allergic phenotypes as well. To navigate the everchanging PID landscape and identify key priorities for the PID community, the International Patient Organisation for Primary Immunodeficiencies (IPOPI) introduced a yearly expert review meeting, the IPOPI Global Multi-Stakeholders’ Summit, during which a select group of PID stakeholders and key opinion leaders join to reflect on current and future challenges and explore (new) opportunities for the entire PID field.

A successful first IPOPI Global Multi-Stakeholders’ Summit held in 2022 was followed by the publication of a set of recommendations concerning; (i) the therapeutic evolution of immunoglobulins (Ig), (ii) personalized management of PIDs, (iii) targeted and curative treatment options, (iv) PID-tailored quality of life (QoL) measures and (v) the known and unknown facets of a constantly evolving PID field and its terminology (1). In line with these recommendations, (i) IPOPI actively engages to improve global Ig availability and access through stakeholder collaboration. This is exemplified, amongst others, by its leading role in the Platform of Plasma Protein Users (PLUS) consortium and the recently produced Memorandum of Understanding between IPOPI and the United Nations Institute for Training and Research (UNITAR), representing a strong shared commitment towards increased access of plasma-derived therapies. Similarly, (ii) through the collaborative initiative “Screen4Rare” IPOPI promotes the widespread implementation of neonatal screening or newborn screening (NBS) to help ensure timely diagnosis and optimized care for rare treatable diseases, including PIDs. In addition, (iii) IPOPI is an active member of the academia-driven “Access to Gene Therapies for Rare Disease” or AGORA group, which sets out to facilitate sustainable access to gene therapies for treatment of patients with ultra-rare diseases by providing centres of excellence with a data repository to support regulatory submissions as well as tailored advice regarding the licensing process (2). Finally, (iv) IPOPI performs research on patient-reported outcomes and experiences in which QoL measures play a central role and (v) IPOPI is assessing the feasibility of a stepwise inclusion of specific actions on secondary immunodeficiencies within the scope of its activities, reflecting the expansion of the immunodeficiency landscape.

The second edition of the IPOPI Global Multi-Stakeholders’ Summit was held on 7 and 8 September 2023 in Cascais, Portugal. Building on the previous discussions regarding sustained access and supply of Ig replacement therapy (IgRT) and its possible alternatives, the first theme of the 2023 summit considered (i) the unmet patient needs towards a more personalized IgRT approach, and the subsequent sessions explored (ii) pandemic preparedness for the PID community and (iii) upcoming tools for improved diagnosis of PIDs. This proceedings’ paper describes the 2023 summit’s outcomes and recommendations.

## Part 1: how can Ig therapy meet future personalized patient needs?

### Enhanced understanding of the mechanism of action of Ig therapy

Plasma-derived IgRT is one of the cornerstones of PID patient management. Despite its widespread use in primary and secondary immunodeficiencies, and the use of Ig therapies for other immunomodulatory indications (e.g., autoimmune and inflammatory disorders) (3), relatively few adaptations have been made to Ig products in the last 50 years (4). While IgRT is marketed under varying formulations, allowing it to be administered via different routes (e.g., intravenous [IV], subcutaneous [SC] and facilitated SC [fSC]), there has been little therapeutic evolution of the immunoglobulin fraction itself (4, 5).

Immunoglobulin products are complex biologically active pharmaceuticals derived primarily from the pooled IgG fractions of thousands of donors, for which no generic counterpart is available. In patients with altered humoral immunity, these polyclonal IgG preparations serve as a mainstay replacement therapy, while their effect on inflammatory and autoimmune disorders is attributed to immunomodulatory and anti-inflammatory properties (6). However, knowledge is still limited on which components of the Ig product are responsible for these multiple immunomodulatory effects, with several specific antibody subfractions having been proposed to modulate different components of the immune system (6, 7). This presents a clear opportunity for more efficacious and efficient product development, which is especially relevant given the high doses of Ig therapy typically needed for treatment of autoimmune and inflammatory disorders (1–2 g/kg of body weight/month) compared to the doses used in replacement therapy for PID and SID (0.4–0.6 g/kg/month) (8). The key issues of plasma shortages, geographical imbalances and dependency in the diverse indications were previously reviewed (1).

While treatment of haemophilia, a rare blood clotting disorder, initially relied heavily on plasma-derived clotting factor replacement therapy, recombinant factor VIII and IX are now commonly used as prophylactic treatment (9, 10). Similarly, experimental results obtained with a modified recombinant IgG crystallizable fragment (Fc) domain in *in vivo* and *ex vivo* models of arthritis (11) and immune-mediated demyelination respectively (12) confirm the potential to create biomimetics with improved effectiveness over classical Ig. Nonetheless, conflicting findings from other studies and human data underscore the need for a better understanding of the mechanism of action of Ig in auto-immune and inflammatory diseases (13–17).

A subpopulation of persons living with antibody deficiencies continues to experience recurrent or persistent airway infections despite appropriate management (18). These patients could potentially benefit from a more targeted administration of Ig products via inhalation. Encouraging results have been achieved in an experimental setting with nebulized immunoglobulins delivered directly to rats’ and non-human primates’ airways (19). This approach is currently under phase I clinical evaluation (20). Alternatively, more personalized Ig therapy in terms of Ig isotype diversity may help address this issue. Currently, most immunoglobulin preparations consist of more than 95% IgG, with only trace amounts of IgA and IgM fractions (21). However, recent studies show that alternative Ig preparations enriched for IgA and/or IgM could be more appropriate and effective for this specific subset of patients (21, 22).

### Emerging infectious diseases and Ig therapy advances

Enrichment of Ig products may also be achieved by the presence of high titres of pathogen-specific antibodies, creating so-called hyperimmune globulins (hIg). Treatment with hIg can significantly reduce the risk or severity of a specific infection, which is particularly relevant in the context of emerging infectious diseases warranting a rapid, targeted approach (23). Typically, hIg are plasma-derived products sourced from selected donors with high Ig titres specific for the pathogen of interest due to natural immunity or immunization (e.g., convalescent plasma from coronavirus disease 2019 (COVID-19) infected individuals) (24). However, efforts are being made in (pre)clinical research towards the development of polyclonal recombinant hIg products (25), as well as monoclonal recombinant therapeutic antibodies derived from human antibody repertoires (26). Recombinant hIg production would help address not only the limitation of plasma availability, but it would also provide a consistent and reproducible product that can be modified for increased efficacy and specificity (27).

Highly specific Ig products would particularly help in the event of local infectious disease outbreaks or for vaccinated individuals experiencing breakthrough infection. Furthermore, due to the geographic variation in endemicity of certain pathogens, persons living with a PID may benefit from receiving IgRT that is sourced from plasma donors living in the same geographical area. Tick-borne encephalitis virus (TBEV) for example is endemic to Central Europe but not the US, resulting in EU-sourced IgRT products having a high TBEV neutralizing capacity while US-sourced IgRT products show no neutralization (28). Currently, however, Europe, like many other regions, is dependent on US-sourced plasma for manufacture of its IgRT products. If limitations in supply and regional legalities would not be a barrier, an even more personalized approach to IgRT could be envisioned based on the individual’s travel plans by administration of products sourced in the region to be visited, prior to travel.

### Additional avenues to explore

Unmet needs and potential for more personalized Ig therapy lies in improved characterization of the relative potency of individual Ig product formulations and their change over time (e.g., due to viral emergence, eradication, vaccination) (29). Similarly, enhanced data collection in the form of registries and other data platforms has the potential to better inform clinicians on a patient’s individual needs. As such, medical monitoring devices and wearables have revolutionized modern healthcare and might be explored to measure overall health status or even specialized parameters (e.g., Ig blood levels, subclinical infections) relevant to PID.

In autoimmunity, alternatives to Ig providing targeted treatment should be considered. As such, the neonatal Fc receptor (FcRn) is now understood to be a crucial potentiator of autoantibody-mediated immune responses, with the first commercial FcRn inhibitor to treat autoimmune diseases such as myasthenia gravis recently having come to market (30, 31).

In addition, curative treatment options such as hematopoietic stem cell transplantation (HSCT) and gene therapy (GT) are increasingly becoming available for PID patients (32–34). Also here, a more personalized approach is recommended to improve HSCT, with the joint Inborn Errors Working Party (IEWP) of the European Society for Blood and Marrow Transplantation (EBMT) and the European Society for Immunodeficiencies (ESID) having developed HSCT guidelines encompassing six different conditioning protocols, tailored to the type of donor and the specific disease background (35). In fact, advances in selected cell depletion (T, B, Naïve T cells…) and graft manipulations now allow for successful allogeneic HSCT even in patients lacking a human leukocyte antigen (HLA)-matched donor (36). Efforts should additionally be focused on advancement of GT approaches, with those in preclinical development exploring *in vivo* – meaning direct in-patient – delivery warranting special attention (37). Critical for the implementation of any GT or other advanced therapy medicinal product (ATMP) will be the creation of an economically sustainable model facilitating development and access, as addressed in the previous IPOPI Global Multi-Stakeholders’ Summit (1).

See **Table 1** for the summary on the prospective (Where do we want to be? And how are we going to get there)?

**Table 1:** How can Ig therapy meet future personalized patient needs?

Where do we want to be?	How are we going to get there?
• Tailored treatment options for autoimmune and inflammatory disorders to help balance the growing Ig therapy demand and consequent supply tensions. • Targeted treatment approaches in emerging infectious diseases. • Locally sourced Ig product formulations providing appropriate protection for common infections in different geographical locations. • Smart use of individual patient data in PID care management. • Tailored treatment of PID patients with recurrent infections and bronchiectasis.	• Increased research efforts towards improved understanding of the mechanism of action underlying successful Ig therapy for autoimmune and inflammatory disorders to allow the production of effective recombinant Ig products. • Development of specific hyperimmune globulin preparations and other therapies (e.g., monoclonal antibodies) for passive immunization. • Development of best practice recommendations and guidelines to help increase regionally balanced plasma collection in an efficacious manner (e.g., enhanced yield/plasma volume). • Personalized data collection through medical monitoring devices and wearables combined with increased integration of available (multi-omics) data into everyday clinical practice. • Investigate the potential of IgA and/or IgM enriched Ig preparations.

## Part 2: pandemic preparedness: what’s next for public health and potential challenges for the PID community?

The risk of emerging infectious diseases (EID) with pandemic potential is higher than ever despite continuous progress in infectious disease control measures facilitated by modern technology (38). Of these emerging pathogens, 60 to 75% are of zoonotic origin, meaning they can be transmitted from animals to humans (39, 40). The recent COVID-19 pandemic, caused by the severe acute respiratory syndrome coronavirus 2 (SARS-CoV-2) which is believed to have arisen from at least two separate zoonotic transmission events (41), clearly demonstrates the significance of EID as a major threat to global health. The increased emergence and spread of zoonoses has been attributed to changes in human and animal interactions, and their surrounding ecosystem.

In particular, global population growth and consequent urbanization, deforestation, agricultural expansion and intensification as well as the trafficking and consumption of wild animals have led to increased interspecies contact and a higher spillover risk (42, 43). The spread of these pathogens is greatly facilitated by fast-growing international trade in meat and other animal products as well as human travel and population movements (44). This alarming situation is further exacerbated by direct negative effects of climate change. Higher temperatures and altered environmental conditions may, amongst others, affect the geographical range of pathogens and their vectors and reservoir hosts, alter pathogen transmission patterns, and contribute to pathogen evolution causing increased virulence (45). Climatic hazards may also render humans more vulnerable to EID by inducing increased bodily stress (e.g., air pollution) or by their indirect impact on food and water security (44, 45). Global efforts to predict, monitor and contain the emergence of new infectious diseases using an integrative approach that considers the interrelatedness of humans, animals and their environment, referred to as “One Health”, are underway (38, 46). Nevertheless, recent estimates of the probability of extreme epidemic occurrence reach a threefold increase in the coming decades (47). Special attention should thus be given to improve preparedness for immunodeficient patients and other vulnerable populations. Identification of high risk PID patients with, for example, additional comorbidities, use of immunosuppressive drugs and other factors such as immune ageing is warranted.

### Crisis and care management during COVID-19: lessons learned

While the collective response to the COVID-19 pandemic has been heavily debated and reviewed, a critical need remains to analyse the crisis and care management strategies applied specifically to PID and other immunocompromised patients. Flexible societies and adaptive governance have proven to be key for efficient viral control and containment (48, 49). This includes dynamic but timely, appropriate and coherent communication to the general public and vulnerable populations (50, 51). Especially the latter received insufficient information tailored to their specific needs and concerns, which greatly worsened the existing feelings of fear and anxiety. Furthermore, current regulations, such as the General Data Protection Regulation (GDPR), pose challenges in maintaining patient lists for swift and targeted communication with specific patient groups. There is limited knowledge and evaluation of effective public health communication strategies targeting high-risk populations in any emergency situation (52). Important lessons should therefore be learned from the recent COVID-19 experience not only on specific considerations and measures to be implemented promptly for immunocompromised patients (e.g., home-based therapy), but also on how to convey such information in an effective and appropriate manner.

Secondly, a lack in flexibility was also evident from the organization of clinical trials investigating vaccines or novel treatment approaches against COVID-19, which did not sufficiently address immunocompromised populations (53). Indeed, despite their high susceptibility to infections, immunocompromised patients were either excluded or poorly represented in the majority of clinical trials investigating COVID-19 vaccines and therapeutics (54). Consequently, the current guidelines for these patients are based mostly on trials conducted in immunocompetent individuals or on real-world evidence collected from use in clinical practice. Yet, suboptimal clinical management of immunocompromised patients infected with SARS-CoV-2 not only undermines their recovery, but also increases the risk of viral persistence which is believed to underlie the emergence of new viral variants which may be less sensitive to treatment (55). There is thus an urgent need for implementation of new adaptive clinical trial frameworks that allow the evaluation of multiple experimental therapies in one or multiple patient subgroups, under one overarching protocol (56). Such novel models, called master protocols, are particularly useful for the study of rare diseases because they typically require only a limited sample size for effective identification of successful therapies (57). In addition, specific trials dedicated to the paediatric population need to be developed as the most severe forms of PID manifest already in early childhood.

### Pandemic control measures for the PID community: going the extra mile

As discussed above, in the early stages of disease detection it is critical to provide clear and specific guidelines for high-risk populations such as immunocompromised patients concerning non-pharmaceutical countermeasures including the wearing of masks and practicing social distancing. Once the disease-causing pathogen has been identified, immunocompromised patients should be prioritized for vaccination, if available, according to medical and scientific up-to-date recommendations. Results from the recent COVID-19 pandemic have shown that mRNA vaccines are safe and effective for most PID patients – with increased immunogenicity compared to classical vaccines – and should thus, at least for SARS-CoV-2, be considered the strategy of choice for this population (58). However, enhanced research efforts are needed to better characterize the induced immune response and lymphocyte subset involvement specific to different types of PID and other immunodeficiencies (59, 60).

Both prophylactic and therapeutic use of monoclonal antibodies and other forms of passive immunization should be explored and implemented. During the early stages of an epidemic, immunocompetent (clinical trial) vaccine recipients could for example be considered as donors for plasma-derived hIg collection. Additionally, efforts should be made to develop direct antimicrobial pre-exposure prophylaxis (PrEP) and post-exposure prophylaxis (PEP) therapy, as is available for human immunodeficiency virus (HIV) (61). Lastly, given the recent advances in chimeric antigen receptor (CAR)-T cell therapy by which genetically modified T cells recognize cell-specific receptors (e.g., tumour-specific receptors), this approach could potentially be exploited to target specific pathogens as well. As such, it would provide an alternative for the management of infections in immunocompromised individuals, particularly when there are no or few drug options. While current CAR-T cell therapies require isolation of autologous T cells, novel strategies using allogeneic T cells derived from donors are under investigation which could broaden the scope of application while reducing its costs (62).

### Antimicrobial resistance: the next threat for the world and even more so for the PID community

Next to the direct challenges related to infectious diseases, a so-called “silent pandemic” of antimicrobial resistance (AMR) has emerged that significantly impacts global public health. AMR, defined as the ability of micro-organisms to survive or grow despite the presence of a previously effective antimicrobial agent, can occur in any type of micro-organism but is most critical in bacteria. It is estimated that in 2019 antibiotic resistance caused 1.27 million deaths worldwide (63). The global economic burden in healthcare costs caused by AMR is estimated to amount to $300 billion to $1 trillion by 2050 (64). The World Health Organization (WHO) has declared AMR as one of the top priorities and global public health threats facing humanity (65), and has recently published a global research agenda prioritizing 40 research topics for AMR evidence generation (66). The issue is particularly concerning to PID patients, of whom a significant proportion is critically dependent on antibiotics and other antimicrobial agents for effective infection management.

Despite this reliance and higher usage of antimicrobials as both prophylactic therapy and acute treatment in the PID population, the presence of AMR in PID – or other immunocompromised patients – is poorly characterized. Recent data show that AMR is common in antibody deficient patients (67) and higher levels of AMR were found for bacterial isolates obtained from PID patients compared to those of immunocompetent individuals, for several antibiotics across multiple types of bacteria (68). This could be explained by the higher frequency and longer duration of antibiotic administration typical for PID patients (68, 69). However, more research is needed on the risk of resistance for individual antibiotics commonly used in PID management, as some long-term antibiotic regimens do not seem to be associated with higher levels of AMR (70, 71). Direct use of IgRT rather than initial prophylactic antibiotic therapy has been suggested for certain primary antibody deficiencies (PAD) (72), as controlled studies showing a benefit of antibiotic prophylaxis in PAD are scarce (73). Some PID medical centres follow a rotational protocol in which antibiotics for prophylactic use are replaced by another agent with equal coverage every 1–6 months to reduce the development of AMR (68, 69, 73). There is however no evidence in the literature supporting the effectiveness of this practice. Similarly, there is a discrepancy in the prophylactic protocols followed for PID patients in a paediatric and in an adult care setting, highlighting once again the need for more streamlined and evidence-based approaches.

Critical to limit the spread of AMR are infection prevention and control measures including environmental disinfection, hand hygiene, isolation and transmission-based precautions. Generally, home-based therapy is preferred for PID patients whenever feasible to avoid contact with hospital-related resistant pathogens. Overall, there is a clear need for improved AMR surveillance as well as increased awareness of infection prevention and control among PID caregivers and antibiotic stewardship to safeguard the effective use of existing antibiotics.

See **Table 2** for the summary on the prospective (Where do we want to be? And how are we going to get there)?

**Table 2:** Pandemic preparedness: what’s next for public health and potential challenges for the PID community?

Where do we want to be?	How are we going to get there?
• Containment and mitigation of the emergence of infectious diseases, with a particular focus on zoonotic diseases. • Inclusion and consideration of immunocompromised patients in crisis and clinical care strategies. • Effective vaccination and therapy schemes tailored to the individual needs of PID and other immunocompromised patients. • Evidence-based protocols for antibiotics and other antimicrobial agents used in PID patients. • Comprehensive surveillance data for infectious pathogens and AMR and global containment and reduction of AMR in the general population.	• Strengthening of the “One Health” approach, addressing root causes (human-animal interactions, ecosystem changes), and improving preparedness for vulnerable populations. • Timely, dynamic and transparent communication tailored to specific high-risk populations, and their inclusion early in clinical trial development. • Increased research efforts exploring the host-pathogen interaction for specific patient subgroups and consequent development of diverse prevention and treatment approaches. • Increased research efforts exploring prophylactic and therapeutic use of antibiotics and the development of AMR in PID patients. • International agreement on data collection and standardization, and increased AMR awareness and antibiotic stewardship as well as improved infection prevention and control.

## Part 3: diagnosing PIDs in 2030: what needs to happen to diagnose better and to diagnose more?

### Tackling the diverging diagnostic rates between countries worldwide

The International Union of Immunological Societies (IUIS) Expert committee’s most recent classification, published in 2022, includes over 485 IEI (74). Diagnosing these disorders is inherently challenging due to the diverse set of clinical presentations and immunological pathways that may be affected. Compounded with the rarity of individual disorders, this often leads to long diagnostic delays (75). In low- and middle-income countries (LMICs), the limited availability of laboratory resources further constraints diagnostic capacities, hindering optimal patient care and prognostic counselling (76). However, increased use and integration of existing and available non-immunological laboratory investigations as screening tools may help identify probable cases for further investigation.

A commonly available and affordable diagnostic test utilized in evaluating routine liver function is calculated globulin (CG), derived from the difference between the total serum protein and the albumin fraction. While abnormally high CG levels may indicate autoimmune disease, infections or cancers, abnormally low CG levels may be caused by decreased serum Ig levels associated with antibody deficiency (77). Importantly, both primary antibody deficiencies, the most common types of PID, and secondary antibody deficiency can be detected using this approach. The test has been validated in multiple centres in both adult and paediatric patients, showing a high sensitivity and specificity for detection of low serum IgG levels, and has allowed identification of patients that were later diagnosed with common variable immunodeficiency (CVID) (77–80). A predictive screening model for the likelihood of hypogammaglobulinemia in the paediatric population has been proposed (81).

These screening tools should therefore be disseminated universally, accompanied by an automated flagging system for further referral to a tertiary care hospital. However, caution is advised in defining clear indicators for referral to prevent overwhelming the tertiary care system.

Considering the complex immunological pathways affected in diverse PIDs, achieving a diagnosis based on molecular and/or functional criteria often demands precise and specific tests. However, the rarity of these disorders poses a challenge to the widespread adoption of such testing methods. High development costs coupled with their limited utilization renders these diagnostic tests economically unfeasible for private or commercial applications. Consequently, such tests are predominantly conducted in research settings, limiting their scope and availability in terms of turnaround time and test variety. Moreover, these research settings are predominantly found in high-income countries (HIC), introducing logistical and financial challenges related to the shipment of viable biological samples from LMICs (82).

In contrast, the recent advancements in genetic sequencing and its decreasing cost have made genetic diagnostics a more viable option for LMIC. While the necessary facilities to perform genetic testing may not be available directly in the geographic region, the inherent stability of DNA allows for the use of dried blood spots collected on filter paper (Guthrie cards) for convenient and cost-effective storing and shipping of high-quality DNA material (83, 84). Next generation sequencing (NGS) approaches have not only facilitated the identification of novel causative variants for PIDs but have also enhanced diagnostic rates (85). Paired with continuous improvement in available genomic databases and bioinformatics, genetic sequencing is therefore envisioned to significantly improve access to diagnosis and care for PID patients in resource-limited settings (82). An important consequence of the enhanced availability and use of genetic testing, however, is the frequent identification of variants of uncertain significance (VUS). Validation of the causal relationship between a new variant and the disease phenotype is often time and labour-intensive, and currently depends largely on the interests and priorities of academic research groups in the field. The introduction of artificial intelligence (AI) and accurate data systems that relate genotypes (variants) to phenotypes may reduce the number of VUS’s (further discussed below) and will contribute to better PID diagnosis. Moreover, AI may be used to study the role of compensating genes and genes causing an exaggerated phenotype. The topic of VUS validation and related recommendations were addressed in greater detail during the previous IPOPI Global Multi-Stakeholders’ Summit (1).

### The role of patient-reported outcome measures (PROM) in improving diagnosis and clinical management

In the past, the assessment of a patient’s progress relied heavily on criteria set by clinicians without substantial consideration for the patient’s experience. However, a significant shift is underway in which the patient’s unique insights and perspectives are increasingly recognized to provide a more comprehensive understanding of the course of the disease and treatment outcomes. In response to this, there is a growing interest in the utilization of Patient-Reported Outcome Measures (PROMs) which record a patient’s perceived health status and well-being in the form of questionnaires or interviews (86, 87). The information obtained through PROMs can serve several purposes at the individual level, including heightened patient engagement in clinical decision-making, personalized care, and continuous monitoring of treatment. Next to their role in clinical management, PROMs are frequently consulted as screening and diagnostic tools in primary care (88). On a broader scale PROMs are valuable tools in the evaluation of clinical research, healthcare service quality, and economic benchmarks (89, 90).

Nonetheless, PROMs are not systematically collected and integrated in healthcare systems. Similarly, despite an increasing trend over time, only a minority of clinical trials and real-world evidence studies include PROMs as primary or secondary endpoints (91, 92). Rare diseases such as PIDs often lack specific and relevant PROMs (93), and patient-reported outcomes are significantly overlooked in paediatric populations (94, 95). Commonly reported barriers to the systematic use of PROMs by clinicians include: (i) time and resource constraints, (ii) insufficient knowledge on questionnaire selection, (iii) data collection and interpretation, (iv) uncertainty on PROM utility and reliability, (v) responder burden for patients, (vi) lack of standardization and integration into clinical workflows, and (vii) the need to develop disease-specific PROM questionnaires (88–90, 96, 97). The latter is particularly problematic for PIDs, as the clinical heterogeneity within this group of disorders prevents the development of useful PID-wide PROMs (93).

Creating an entirely new disease-specific PROM is highly labour-intensive and requires careful analysis and validation before it can be introduced. The Patient-Reported Outcomes Measurement Information System (PROMIS) aims to facilitate this process by producing validated, standardized item banks measuring different aspects of health-related quality of life (HR-QoL), relevant across a range of chronic conditions (98). Based on the selection of those item banks most appropriate to the specific disease in question, a customized and sensitive PROM can be built. While the use of PROMIS item banks for rare conditions overall has increased (99), there is ample room for such measures to be developed and implemented in the PID field. One study has been conducted using the PROMIS-29 score for chronic diseases in CVID patients, which underscored the impact of fatigue on HR-QoL for this patient population (100). Additionally, a PROM specific to CVID patients was developed and validated to be a reliable tool, identifying fatigue and other HR-QoL issues associated especially with CVID (101). However, more efforts are needed to adequately capture non-CVID patients’ self-reported HR-QoL.

Next to PROMs which capture the patient’s view regarding their health status and physical and mental wellbeing, patient-reported experience measures (PREMs) are critical to gather information on the patient’s perception of their received care, reflecting their satisfaction with different aspects of the clinical care provided (102). Patient organisations, in conjunction with clinical specialists, are ideally positioned to advocate for the implementation of both PROMs and PREMs as patient-centred measurements (PCM) in every aspect of clinical care and research. They can help develop qualitative and useful questionnaires tailored to the needs and wishes of their specific patient populations, as is exemplified by the CVID_QoL questionnaire which was produced based on the outcomes of individual and patient group consultations (101). To streamline the collection of PCMs and ensure compliance with data protection regulations, the implementation of digital applications enabling data decentralization coupled with modern technologies driven by AI to process natural language is a viable approach. Such tools allow personal data to be owned by the patient while facilitating the aggregation of PROM and PREM data and establishing mechanisms for secondary use (103–106). Additionally, using this approach patients can report their outcomes in ‘real-time’ which increases the data’s reliability and accuracy (107). If made available to clinicians, these electronic PCMs could complement existing registries to guide medical decision-making and be consulted for large-scale research initiatives. Indeed, the use of PCMs should be promoted as an integral part of “value-based” medicine which aims to maximize patient relevant outcomes at the lowest possible cost, whenever care is provided (108). For it to be successful it is key to understand what matters most to patients, thereby necessitating PCMs to be at the core of this healthcare model (97).

### The role of artificial intelligence (AI) in improving diagnosis

The last decade has witnessed great advancements in the integration of AI into all aspects of medicine. Machine learning algorithms, by which large datasets are iteratively used to extract information allowing the software to gradually improve its performance, can aid in the early detection of conditions by identifying patterns which are impossible for humans to discern (109). As mentioned above, this is especially attractive for the diagnosis of rare diseases, considering that such patients are often confronted with a long diagnostic delay (110, 111). Alternatively, once a certain pattern of characteristics has been linked to a rare disorder, AI, leveraging Natural Language Processing techniques, can be utilized to automatically screen a large number of electronic health records, enabling the reevaluation of patients who may have been undiagnosed in a hospital’s database (112). AI is also a powerful research tool to generate new hypotheses on the genetic or molecular pathogenesis underlying a disease, further improving the diagnostic potential (113).

However, machine learning algorithms require clean, comprehensive datasets to produce correct and precise predictions. Yet, digital data in the field of medicine is often scattered or inaccessible, which is further compounded for rare diseases by their inherently small patient populations generating only limited data (114). It is therefore critical to combine hybrid models (machine learning models, rule-based and knowledge-based reasoning) and diverse types of input data, ranging from clinical and phenotypic descriptions – possibly collected via natural language processing of electronic health records – to laboratory test results, imaging, and high throughput molecular profiling outcomes (e.g., genomics, proteomics, metabolomics, etc.) linked to available knowledge databases (110, 115, 116).

Important hurdles to making this data accessible in real time and in raw format relate to issues of data ownership and privacy, bringing about ethical considerations and regulatory constraints. These limitations also hinder effective collaboration and data sharing between healthcare facilities within and across countries, thereby restricting the integration and harmonization of AI-driven diagnostic tools on a larger scale (117). One approach to overcome these limitations is federated learning, a technique that allows for collaborative machine learning without sharing the actual data, maintaining privacy and compliance with regulations. It enables healthcare providers to update models in real time and improve their generalization by learning from diverse datasets without compromising data security. While promising, implementing federated learning requires careful consideration of technical infrastructure, efficiency, regulatory oversight, and ensuring incentives for participation (118).

Establishing international consensus and guidance within a policy framework will be imperative to address the current challenges related to AI integration in clinical practice and research. To this end, governments and political institutions worldwide have started to develop regulations concerning the use of AI in healthcare, such as the proposed European legal framework “AI Act” for which a political agreement was recently reached (119). To ensure the effectiveness and acceptability of AI tools for broadscale clinical implementation it is crucial to involve all relevant stakeholders throughout their development and integration (120, 121). Patient organizations, along with clinicians, regulators, and other relevant parties, should be actively engaged, as they are ideally positioned to bridge potential gaps between the AI developer and the patient community (120). The ability of AI to transform patient records into computational objects will ensure faster access and smarter use of the large volumes of clinical data being collected, catalysing our diagnostic ability and accelerating translational research and personalized patient care.

See **Table 3** for the summary on the prospective (Where do we want to be? And how are we going to get there)?

**Table 3:** Diagnosing PIDs in 2030: what needs to happen to diagnose better and to diagnose more?

Where do we want to be?	How are we going to get there?
• Universal use and integration of available laboratory investigations as diagnostic screening tools to tackle the diverging diagnosis rates between regions. • Accessibility of genetic diagnostic tools regardless of geographic region. • Systematic integration and use of PROMs in clinical care pathways and clinical research. • Broad-scale integration of AI-based diagnostic tools in healthcare practices and research for PID and the larger rare disease community. • Active involvement of patient organizations and all relevant stakeholders in the development and integration of AI tools.	• Implementation of calculated globulin or other diagnostic tools as screening methods in an automated flagging system, both in high and low-resource settings. • Enhanced international collaboration and smart use of logistically favourable and cost-efficient alternatives as source material (e.g., dried blood spots on filter paper). • Digitalization of PROMs for facilitated collection of real-time patient-reported data and integration in electronic health records and/or registries. • International alignment and enhanced interoperability through robust legal frameworks facilitating data sharing while safeguarding data ethics. • Consider the creation of an IPOPI-led initiative on AI integration in patient diagnosis and other aspects of clinical care.

## Data availability statement

The original contributions presented in the study are included in the article/supplementary material. Further inquiries can be directed to the corresponding author.

## Author contributions

SV: Writing – original draft, Writing – review & editing. JP: Writing – original draft, Writing – review & editing. SS: Writing – review & editing. DL: Writing – review & editing. MB: Writing – review & editing. BA: Writing – review & editing. GS: Writing – review & editing. AP: Writing – review & editing. NG: Writing – review & editing. CB: Writing – review & editing. SS: Writing – review & editing. JD: Writing – review & editing. IQ: Writing – review & editing. IM: Writing – review & editing. AA: Writing – review & editing. SB: Writing – review & editing. Mv: Writing – review & editing. MP: Writing – original draft, Writing – review & editing. NM: Writing – original draft, Writing – review & editing.

## Glossary

**Table:**

AGORA	Access to Gene Therapies for Rare Disease
AI	Artificial Intelligence
AMR	Antimicrobial Resistance
ATMP	Advanced Therapy Medicinal Product
CAR	Chimeric Antigen Receptor
CG	Calculated Globulin
COVID-19	Coronavirus disease 2019
CVID	Common Variable Immunodeficiency
EBMT	European Society for Blood and Marrow Transplantation
EID	Emerging Infectious Diseases
ESID	European Society for Immunodeficiencies
Fc	crystallizable fragment
FcRn	neonatal Fc receptor
GDPR	General Data Protection Regulation
GT	Gene Therapy
HIC	High-Income Countries
HIV	Human Immunodeficiency Virus
hIg	Hyperimmune globulins
HLA	Human Leukocyte Antigen
HSCT	Hematopoietic Stem Cell Transplantation
HR-QoL	Health-related quality of life
IEI	Inborn Errors of Immunity
IEWP	Inborn Errors Working Party
Ig	Immunoglobulin
IgRT	Immunoglobulin Replacement Therapy
IPOPI	International Patient Organisation for Primary Immunodeficiencies
IUIS	International Union of Immunological Societies
IV	Intravenous
LMIC	Low- and Middle-Income Countries
NBS	Newborn Screening
NGS	Next Generation Sequencing
PAD	Primary Antibody Deficiencies
PCM	Patient-Centered Measurement
PEP	Post-Exposure Prophylaxis
PID	Primary Immunodeficiencies
PLUS	Platform of Plasma Protein Users
PREM	Patient-Reported Experience Measures
PrEP	Pre-Exposure Prophylaxis
PROM	Patient-Reported Outcomes Measures
PROMIS	Patient-Reported Outcomes Measurement Information System
QoL	Quality of life
SARS-CoV-2	Severe Acute Respiratory Syndrome Coronavirus 2
SC	Subcutaneous
TBEV	Tick-borne encephalitis virus
UNITAR	United Nations Institute for Training and Research
VUS	Variant of uncertain significance
WHO	World Health Organization
